# Koumiss, and Its Use in Medicine

**Published:** 1874-11

**Authors:** Oscar C. DeWolf

**Affiliations:** Chicago


ARTICLE II. — *Koumiss, and its Use in Medicine.* By OSCAR C. DE WOLF, M.D., Chicago.

During the years 1858, '59, '60, '61, while a student of medicine in England, and on the Continent, I heard occasionally of the wonderful effect of a preparation of fermented milk when taken largely as a dietetic remedy. I recall particularly a clinical lecture by the immortal Trousseau, at the Hotel-Dieu in 1860, in which he confirmed the statement of a German investigator that "it possessed the highest power of nutrition, and was one of the most decided enemies of emaciation we could produce." It does not appear to have received the attention in this country which it merits, and while enthusiasm may (after the fashion of our people) misrepresent it as a cure-all, it nevertheless has a legitimate place in medicine which a more extended acquaintance and use will develop and define.

I have been able to gather much that has been written in Germany and England on the subject, and I venture to collate such facts in regard to its history, and use, as will interest the profession and command their attention. I have consulted the following works, and some of them are now before me. Grieve, in Edinb. Transact. 1787, pp. 278; the London Med. Journal, 1789; Ucke on the Climate and Diseases of Samara, 1863; The Result of Eight Years' Residence in Orenburg, 1843–51, by Dr. P. DeMaydell, Medical Inspector for the Government of Oriel, Russia; Balneologische Notizen uber die Kurmittel des Bades Reinerz, by Dr. Herrman Biegel, physician to the Metropolitan Free Hospital, London, 1863; Koumiss, in the Treatment of Phthisis, by Dr. Jagielski, London, 1872; Der Kumys und edessen Anwendung in der Therapie, by Dr. J. Brzezinski, Berlin, 1872; Der Kumys Seine Physiologischen und Therapeutischen Wirkungen, by Dr. E. Stahlberg, St. Petersburg, 1869; Therapeutical Properties of Koumiss, translated from Le Progress Medical, by Fred. J. Huse, M.D., in Medical Examiner, (Chicago) Oct. 1, 1874. Further acknowledgment for whatever I may use from the above authors is unnecessary.

Dr. Grieve says, "it may appear surprising that one of the most important productions of milk should still remain in a great measure unknown to the most enlightened portions of Europe.

This production, which is procured by fermentation from mare's milk, is a vinous liquor, and it was scarcely to be expected that after it had escaped the observation of men the most skilled in chemistry, it should be taught us by a horde of Tartars, whose rank in the social scale is not above that of barbarians."

Koumiss as prepared from mare's milk, has its native country in those regions of the Russian Empire in Asia, between the Don, Volga, and Sea of Aral, consisting of the Steppes, inhabited by the Tartar tribes, especially in the country of Orenburg, which, according to Von Humbolt, belongs to the so-called "Continental climate," where the summer is exceedingly dry, and the heat very great, with scarcely any rain, whereas in winter, continuous frosts are prevalent. The inhabitants are a nomadic people, generally of a strong and vigorous habit of body, with great muscular development and well shaped thorax. Their food is very simple; they live exclusively on meat, especially the flesh of the sheep and horse, and in winter and summer their favorite beverage is koumiss. In order to obtain milk in sufficient quantity the Tartars are accustomed to separate the foal from the mare during the day, allowing it to suck only at night, and the milk is taken from the mare about five times a day. During the winter when mare's milk fails them they use the milk of the cow, which is also susceptible of the vinous fermentation. The preparation from cow's milk they call arjen or arjan, but koumiss is the Tartar name for the fermented mare's milk. They distil a spirit, highly intoxicant, from both koumiss and arjan, which they call arika, and which they keep all the year round in stock.

The mare's milk is kept in a bag made of horse's hide undressed, which by smoking acquires a degree of hardness. Its shape is conical, somewhat triangular, from being composed of three different pieces set in a circular base of the same material. The sutures, which are made with tendon, are secured by a covering on the outside with a doubling of the same skin, closely fastened. These bags are used both for the preparation and transportation of koumiss, and have a dirty appearance and disagreeable smell. The best koumiss, according to Dr. Stahlburg, is made near Orenburg, while that in the departments of Stauropol, Taurien, and on the Caucasus, is not so well prepared; it has an unpleasant effluvium, as even the best preparations from mare's milk always have more or less, but to which patients soon grow accustomed.

To appreciate the chemical changes which milk undergoes in the process of fermentation, we must fix our attention on the decomposition of milk sugar into alcohol, carbonic acid, and lactic acid.

Milk sugar is said to be composed of "$C^{12}$ $H^{22}$ $O^{11}$ + $H^2$ O; by the addition of a ferment it decomposes into C $O^2$, and alcohol $C^2$ $H^6$. In consequence of this decomposition the sugar yields two bodies: 1. Basic alcohol; 2. Carbonic and lactic acids. While the milk sugar alone is not fermentable, it is capable of conversion into a grape sugar which is fermentable." (Lebert.)

The principal ingredients of milk are casein, fat, milk sugar, mineral salts, especially phosphate of lime, chlorate of potass, magnesia, some iron, etc., and water. The proportion in which these ingredients occur in milk is not only a variable one in man and in different animals, but varies also, and in certain cases very much, in one and the same individual.

According to Dr. With, woman's milk is proportionately poor in cheesy matter, but rich in milk sugar, and fat. Mare's milk is still poorer in cheese, very poor in fat, but extremely rich in sugar. Goat's milk, and that of the cow, are very similar in their proportion of casein, fat, and milk sugar.

The following table is interesting as a matter of reference:

"The milk of all animals is converted into koumiss by one and the same method," but it will be seen from the above that the milk of the mare, so rich in sugar, will furnish a larger per cent. of alcohol; yet the cow's milk may be approximated by the addition of four per cent. of sugar and two per cent. of fat.

In considering the physiological effect of koumiss, we may suppose that in addition to the well known nutritious value of animal milk, there are certain accessory agents produced by the fermentative process, to wit: Alcohol, carbonic and lactic acids; and, still further, that the casein, particularly of the cow's milk, when used, has undergone such an important modification that it is dispersed through the fluid in loose floculent masses, " which develop bubbles so long as they are moist, and which dissolve on the tongue like snow."

Three grades of koumiss are manufactured in Germany and Russia, known as A, B and C. " Full koumiss (A) is made from the cow's milk, preserving the whole of the cheesy matter. Medium koumiss (B) is the artificial representant, as nearly as possible, of that made from the mare's milk, by withdrawing the proper quantity of casein and fat, and the addition of such a per cent. of sugar of milk as will approximate the cow's milk to that of the mare. In whey koumiss (C) the casein is entirely removed." By proper manipulation, it is claimed that " the milk of the cow is an equally good, if not better, raw material as that of the mare," and it certainly affords a facility for varying the composition of the product, that the lighter milk of the mare does not possess. From the commencement of fermentation to its completion, that is, until the sugar has been entirely broken up into alcohol, each grade passes through three degrees of fermentation, and when once commenced it will continue, no matter what the temperature, but in passing through these changes it retains all of its general and remedial properties. " In all cases it is a milk-like fluid. In its fresh (No. 1) state it is a still liquor, and has an agreeable, sweetly-acidulous taste. As it approaches No. 2 it becomes more acidulous, and assumes a sparkling character. In No. 3 the sweet taste is replaced wholly by the acidulous, slightly bitter, and it not only sparkles, but rushes from the bottle with great force."

Passing now to the therapeutical uses of " milk wine," it would be useless to review the great mass of clinical testimony presented by the authors to whom I have referred.

The public at large in Russia is accustomed to regard koumiss as a specific against tuberculous affections, and Dr. Stahlburg declares, that " if a remedy against phthisis exists in the world, it *must be* koumiss. Seeland (Medicin der Gegenwart, January, 1862) compares the effect of koumiss to that which is produced by transfusion, and claims that medicine is in possession of no blood restorer to be compared to it.

Allowing the widest margin for the enthusiasm of the German and French investigators, the legitimate and rational indications for its use are apparent to the practical physician. It cannot be a specific in phthisis, but it may be of immense service in counteracting the disturbed and degraded nutritive changes which predispose to it, while it certainly supplies a food peculiarly adapted to conditions of exhausted vitality, emaciation from any cause, and the gastro-intestinal affections of children. In brief, it must be accorded a high place in the series of plastic nourishments.

My own experience in its use has been very limited, yet small as it is, has been interesting, and is, I trust, worthy of brief notice in connection with this paper.

After many trials and much patient labor, Mr. Arend, druggist, 521 West Madison street, succeeded in producing a koumiss which, so far as I can judge, is a good article. It answers, in every particular, to the description given by those who are expert in its manufacture and use.

While he was experimenting, and not yet satisfied with his product, I was called, on the evening of July 25, to visit a child twenty-two months old, two days sick with cholera infantum. Neither food, medicine, nor ice had been retained in the stomach a moment for eight hours; the dejections were frequent and characteristic; surface cool; face pinched; and the fearful screech every two or three minutes was indicative enough of the complete nervous exhaustion and approaching death of the patient.

I had veryrecently read Dr. Jagielski's paper on koumiss, read before the London Medical Society, in Feb., 1874. In it he dwells much on the magical effect of koumiss, highly charged as it is with carbonic acid, in allaying nausea, calming gastric irritation, and increasing the energy of the heart's impulse. I found a beer bottle of full (A) koumiss at Mr. Arend's, five days old, and returned to my patient.

In opening the bottle, two-thirds of it escaped, but enough was secured to make a beginning, and the child took two tablespoons-full, a greater part of which was retained. A second bottle was procured. Some of the casein, which had separated in floculent masses, was taken out, and the child fed freely with the remaining liquor. She took no other food, fluid, or medicine. In three hours she slept, the surface became warm, discharges gradually ceased, and she steadily convalesced.

I frankly confess my belief, that I had no other than this accidental resource which would have successfully met the emergency.

Mr. F. H. L., aet. 26, clerk in banking house in this city, of a consumptive family, consulted me in March last. I could detect no local pulmonary trouble, yet his general condition was far from satisfactory. I advised him to eat fatty food, dress warmly in flannels, and change his occupation. From time to time he reported himself as improving, and so it seemed. On July 27, while at his desk, he had an attack of violent hæmoptysis, which was repeated in three days. I only report his progress and so much of his case as is of interest in connection with the present subject. The lower two-thirds of right lung became solid; not the slightest cough nor expectoration; stomach irritable; would take neither milk, champagne, nor alcoholic stimuli of any kind; slept badly; pulse, 115–30; heat of surface, 101°–3°; emaciation rapidly progressive.

Towards the last of August he became so feeble that he could only be moved from side to side of the bed with much care, and always in a horizontal position. His family were summoned from Connecticut, and I had no thought of his living a week. Prof. Henry M. Lyman, who had seen the patient twice during his illness, was again desired to visit him, and confirmed my opinion of a probable fatal result soon; that he could not be moved, etc. The koumiss was now in successful manufacture at Mr. Arend's, and on August 28th he commenced its use.

He had great disgust of milk, could never take a tablespoonful without pain following it, and objected strongly to the koumiss. He soon learned to love it, however, and took, for ten days, about one quart per day. His sleep returned: indeed, his nurse and friends were fearful he was sleeping too much, and would awaken him; pulse slowed to 100 per minute; temperature fell to 99°; and his strength was so far recuperated that, on September 10th, he was ready to undertake a journey of fifteen hundred miles, which he accomplished comfortably. Since deprived of his koumiss, he is again sinking, and in a letter from his father, dated September 23d, he informs me: " Frank has gone back about where he was before he commenced the koumiss." While the case would have progressed to a fatal issue under any circumstances, yet the markedly restorative influence of koumiss was well exhibited, and were my paper not already too long, it would be instructive to consider the "manner and the method" of its help to my patient.

In a case of carcinoma of the rectum, in which the patient had become greatly exhausted from the profuse discharge, and an irritability of the stomach, which for days refused all nourishment, the effect of koumiss in arresting vomiting, producing sleep, and temporarily reviving the strength, was extremely gratifying. Prof. J. Adams Allen saw this lady, in consultation.

Mr. B., æt. 31 years, bookkeeper, has suffered much from dyspepsia and nervous depression for the past two years, and for three months, last spring, was not able to occupy his desk. Food distressed him; bowels distended and sore; considerable emaciation. In July he visited the Atlantic coast to obtain benefit from the sea air. Not much improvement. Commenced taking koumiss last of August, and in one month had gained in weight seven pounds; called himself well. Early in October he returned to his ordinary diet, and his dyspeptic troubles recurred. He is now taking koumiss, with the same benefit as at first.

The quantity of koumiss advisable to take in commencing its use, should not exceed one champagne bottle daily, for an adult, and even that amount sometimes produces a slight febrile reaction. If headache occurs, it should be suspended for a day or two; but in a short time a toleration is generally established, and the patient soon discovers how much he can take, and then he may live entirely upon koumiss, or, when the stomach will tolerate other food, it may be added. It does not interfere with any other course of treatment. The secretion of urine is generally much increased. When a tendency to constipation exists, the fresh product (No. 1 or 2) should be used, but if diarrhœa is troublesome, an older sort should be taken. A piece of stale bread or cracker, eaten immediately after drinking, will relieve the fullness of stomach sometimes complained of.

The desire for sleep generally experienced after drinking koumiss, should be regarded as beneficial, and should not be interfered with.

